# Mapping the Lay of the Land: Using Interactive Network Analytic Tools for Collaboration in Rural Cancer Prevention and Control

**DOI:** 10.1158/1055-9965.EPI-21-1446

**Published:** 2022-04-20

**Authors:** Bobbi J. Carothers, Peg Allen, Callie Walsh-Bailey, Dixie Duncan, Rebeca Vanderburg Pacheco, Karen R. White, Debra Jeckstadt, Edward Tsai, Ross C. Brownson

**Affiliations:** 1 Center for Public Health Systems Science, Brown School, Washington University in St. Louis, St. Louis, Missouri.; 2 Prevention Research Center, Brown School, Washington University in St. Louis, St. Louis, Missouri.; 3 Butler County Community Resource Council, Poplar Bluff, Missouri.; 4 Missouri Highlands Health Care, Ellington, Missouri.; 5 Missouri Ozarks Community Health, Ava, Missouri.; 6 Division of Public Health Sciences, Department of Surgery, Washington University School of Medicine, Washington University in St. Louis, St. Louis, Missouri.; 7 Alvin J. Siteman Cancer Center, Washington University School of Medicine, Washington University in St. Louis, St. Louis, Missouri.

## Abstract

**Background:**

Cancer mortality rates in the United States are higher in rural than urban areas, especially for colorectal cancer. Modifiable cancer risks (e.g., tobacco use, obesity) are more prevalent among U.S. rural than urban residents. Social network analyses are common, yet rural informal collaborative networks for cancer prevention and control and practitioner uses of network findings are less well understood.

**Methods:**

In five service areas in rural Missouri and Illinois, we conducted a network survey of informal multisector networks among agencies that address cancer risk (*N* = 152 individuals). The survey asked about contact, collaborative activities, and referrals. We calculated descriptive network statistics and disseminated network visualizations with rural agencies through infographics and interactive Network Navigator platforms. We also collected feedback on uses of network findings from agency staff (*N* = 14).

**Results:**

Service areas had more connections (average degree) for exchanging information than for more time-intensive collaborative activities of co-developing and sustaining ongoing services and programs, and co-developing and sharing resources. On average, collaborative activities were not dependent on just a few agencies to bridge gaps to hold networks together. Users found the network images and information useful for identifying gaps, planning which relationships to establish or enhance to strengthen certain collaborative activities and cross-referrals, and showing network strengths to current and potential funders.

**Conclusions:**

Rural informal cancer prevention and control networks in this study are highly connected and largely decentralized.

**Impact:**

Disseminating network findings help ensure usefulness to rural health and social service practitioners who address cancer risks.

## Introduction

Rural areas in the United States have higher incidence rates than urban areas of several types of cancer with modifiable risks, including cancers of the lung and bronchus, cervix, and colorectal cancer (CRC; refs. 1–3). Five-year mortality rates for any type of cancer in the United States are 182 per 100,000 in nonmetropolitan counties and 166 per 100,000 in metropolitan counties (1, 2), and higher for colorectal cancer specifically (4). Internationally, cancer screening rates are lower in rural areas overall and for colorectal cancer (5). U.S. rural areas have greater proportions of households in poverty and uninsured adults, affecting access to screening (6). Human papillomavirus (HPV) vaccination rates are lower in U.S. rural areas (7, 8), as are cervical cancer screening and treatment rates (7). Modifiable cancer risk factors affecting excess rural cancer burden include tobacco use, physical inactivity, nutrition patterns, obesity, and heavy alcohol use, each of which is higher in U.S. rural than urban areas (9–14). Obesogenic environments (15) and food insecurity (16) are more commonly found in rural counties than in micropolitan or metropolitan counties in the U.S. Rural adults report higher intake of sweetened beverages and potatoes, and lower intake of fruits, green vegetables, and fiber than urban adults (17, 18).

Multisector collaboration, or cross-sector collaboration, involves the coordinated efforts across multiple governmental agencies, public and private organizations, and/or community groups (19). Multisector collaboration is a widely promoted strategy (20–22) that can improve access to services (23), use of services including cancer screening (24), health behaviors (25, 26), and health outcomes (27, 28). For example, policy and built environment changes from multisector collaborations increase smoke-free environments (26) and places for safe physical activity (29).

Informal collaborative networks are increasingly common networks that arise to address complex community problems (30–32). Such networks aim to connect public, nonprofit, and for-profit agencies across sectors to improve delivery of services and interventions at multiple levels and settings to address difficult issues. Informal networks often have weak or diffuse oversight and blend resources from a variety of sources, each having its own stipulations for service or program delivery (30, 33). Although informal networks are common in prevention, they are less well-studied than formal grant-funded networks or policy networks (30), and even less commonly studied in rural areas (34), where organizations link with fewer agencies than in urban areas (34). Informal networks can benefit from network visualizations and analyses that demonstrate network structures, strengths, and gaps (30, 35), yet we found little in the literature on how best to disseminate social network analysis findings to optimize usefulness to collaborating agencies.

Despite increased attention to multisector collaboration in metropolitan areas, less is known about the nature and effectiveness of such collaborations in rural communities, especially informal networks. The purposes of the present study are to: (i) explore multisector collaboration networks for cancer prevention in selected rural low income service areas; and (ii) describe how rural agencies use network information to strengthen their interagency networks and intra-agency processes. The current study is part of a larger project that also sought to identify implementation capacity and the extent of implementation of evidence-based cancer prevention interventions in rural southeastern Missouri and southernmost Illinois.

## Materials and Methods

The study team developed and conducted a network survey informed by key informant interviews and prior work, then examined the data with social network analysis (SNA) and visualization methods. We disseminated network findings through summary infographics and an interactive Network Navigator platform. The Institutional Review Board of Washington University in St. Louis (St. Louis, MO) approved the human subjects study with exempt status in accordance with the Belmont Report.

### Participants/data collection

Development of the network survey was informed by 32 key informant interviews conducted from February to March 2020 (*n* = 13) and July to August 2020 (*n* = 19) with staff from Federally Qualified Health Centers (FQHC; community health centers that provide primary and behavioral health care to low-income patients), local public health departments (LHD), schools, and community partners (e.g., social service agencies, faith-based organizations, local governments, food pantries) in four FQHC service areas in rural Missouri and seven rural counties in Illinois served by a single LHD (36). Each Missouri FQHC service area covered 4 to 7 counties. We used a combination of purposive and snowball sampling approaches. In each service area, we selected one high resource/lower need county and one low resource/high need county to focus interviews. High-need counties were those defined as having cancer risk higher than the state average and higher than average risk (poverty, physical inactivity, lack of fruit and vegetable intake, fat intake, tobacco use, heavy alcohol use, lack of cancer screening and high all-cancer mortality; refs. 37–40) for the service area. Number of LHD employee full time equivalents per jurisdiction population was a proxy measure for resources to address cancer risk (41). Within service areas, participants suggested contacts within their agency or other partner agencies to contact for additional interviews. Interview participants described interagency collaboration activities for cancer prevention and detection to increase access to and promote physical activity, healthy eating, tobacco use prevention and cessation, HPV vaccination, and screening for colorectal, breast, cervical, and lung cancers. A thematic analysis approach was used to elicit activity types for network survey items (42). From these interviews, we learned the cancer-control activities that agencies collaborated on, key agencies to include in those service area networks, and which individuals should represent those agencies.

Informal collaborative interagency networks in the four FQHC service areas in Missouri and a multiple-county LHD service area in Illinois participated in the network survey, ranging in size from 24 to 45 agencies. Agencies included those mentioned above, as well as university extensions and healthcare facilities (e.g., hospitals, medical centers). We sent a Qualtrics (43) web-based survey to agency contacts asking about their relationships with other agencies in their service area network. The survey ran from late September through mid-December 2020. Participants were offered a $20 Amazon gift card.

Network maps were disseminated via an infographic summarizing findings from their own service area's network, as well as an interactive network application for key agencies that expressed interest. Uses of the network findings were collected from participants who were highly engaged during the dissemination phase.

### Measures

Given the impact of the COVID-19 pandemic starting in March 2020, we asked participants to answer for their relationships as they were during calendar year 2019 to get a snapshot of their pre-COVID connections for cancer prevention or detection. We measured relationships for contact frequency, collaboration on five activity types, and referrals. A template of the survey document is provided in the Supplementary Methods and Materials.

Uses of network findings were collected in two ways. Informal feedback was provided by 14 dissemination session attendees in nine separate dissemination sessions. Formal written feedback was invited from a purposive sample of agency staff who made use of the interactive network application. They responded via email to open-ended questions about which visualizations were most useful, how they planned to use the network information, what barriers they foresaw or encountered in using what they learned, any recommendations they had for other practitioners on using network information and for researchers on conducting network research, and any improvements they would like to see on the interactive network application.

### Network data management

When more than one individual responded for an agency, network relationships were aggregated to the agency level such that: (i) the highest value for contact was selected, (ii) any participation of activities was accepted, and (iii) any selection of referrals was accepted (except for “Neither”).

Because contact is theoretically a nondirected relationship (if agency A said they were in contact with agency B on a monthly basis, B should say the same about A), values for yearly through weekly were symmetrized using the lower of the two values indicated by each pair so as to not overestimate the relationship. If only one agency of the pair responded yearly or more, the value of the responding agency was used. Contact could then be examined at four different levels: at least weekly, at least monthly, at least quarterly, and at least yearly.

Activities were nondirected relationships–if agency A said they developed and shared resources with agency B, B should say the same about A, so links between pairs were symmetrized such that a link between A and B was considered to exist if either or both indicated working together on it. Referrals were a directed relationship–if agency A sent referrals to agency B, B didn't necessarily send referrals to A. A referral from A → B was considered to exist if A indicated sending referrals to B and/or if B indicated receiving referrals from A. A bidirectional relationship (A ←→B) was considered to exist if both indicated sending referrals to or receiving referrals from the other, or one or both indicated both sending and receiving referrals.

### Analysis

Node (agency) level statistics were calculated for the nondirected relationships (contact and activities). Degree is the number of agencies an agency was connected to. Agencies with high degree can reach many other agencies directly. Betweenness centrality is the extent to which an agency is on the paths that link all of the other agencies in the network, and can be thought of as the extent to which it connects agencies that are not otherwise connected. Agencies with high betweenness centrality have a great deal of control over exchange in the network. For referrals, a directed relationship, in-degree (the number of incoming links) and out-degree (the number of outgoing links) were calculated.

Network-level statistics were also calculated. Average degree is the average number of connections for the agencies in the network. Degree centralization is the extent to which the network has one or a few agencies with many connections and ranges from 0 to 1. In-degree, out-degree, and total-degree centralization can be calculated for directed networks. Betweenness centralization is the extent to which the network has one or a few agencies that keep the network connected, also ranges from 0 to 1, and was only calculated for nondirected networks. See Wasserman & Faust (1994) for more details (44). Statistics were calculated with R igraph (v 1.2.8).

### Dissemination

All survey participants received an infographic summarizing findings from their own service area's network survey. Key agencies were offered password-protected interactive network applications for their own networks that displayed visualizations and network-level statistics for all relationships and a video conference session orientation to the interactive network application. (See https://netnav.shinyapps.io/demonet/ for a generic demonstration version of the interactive Network Navigator application.) The application developed for this project provided a brief introduction on how to interpret network maps and statistics and allowed users to explore the networks directly. Users could choose which levels of contact to display; whether to size nodes by degree, betweenness centrality, or equally; and so on. Clicking on individual nodes displayed degree and betweenness centrality statistics for that agency and how it compared with the network average. Network-level statistics were provided in a table below the map. The applications were built in the R Shiny environment using the R visNetwork package (v 2.0.9) for map visualizations. Users could download their network maps, agency-level, and network level statistics, and were offered individualized demonstrations of the network application by study staff.

### Data availability

Deidentified network data in the form of igraph objects for each relationship are available in an Rdata file upon request to the corresponding author.

## Results

### Participants

Of 182 individuals representing the 158 invited organizations across the five service areas, 152 completed surveys (83.5% individual response rate overall, ranging from 82.1% to 85.7%). Agency response rates ranged from 86.7% to 92.8% over the five service areas. The number of agencies included in a service area's survey ranged from 24 in the lowest population service areas to 42 agencies (Table 1; ref. 45). All service areas had one FQHC except for Area 5, which had two.

**Table 1.** Service area characteristics.

Service area	Number of agencies in network survey	Number of counties	Area population^a^	LHD employee FTEs per 1,000 area population^b^
Area 1	30	4	138,957	0.58
Area 2	42	7	100,713	0.87
Area 3	24	4	66,574	0.56
Area 4	38	6	147,771	0.45
Area 5	24	7	64,560	0.56

Abbreviation: FTE, full time equivalents.

^a^U.S. Census Bureau. 2018 American Community Survey.

^b^National Association of County and City Health Officials. 2016 National Profile of Local Health Departments. Total number of local health department employee FTEs divided by total service area population. FTE/area population is a proxy measure for prevention resources.

### Collaborative activities

The survey asked about five types of collaborative interagency activities: exchanging general information, promoting each other's services and programs, cohosting annual or one-time awareness events, co-developing and sustaining ongoing services and programs, co-developing and sharing resources; as well as referrals to and from each other. Table 2 shows that overall, the five service areas had greater numbers of connections (average degree) for exchanging information than for more time-intensive collaborative activities of co-developing and sustaining ongoing services and programs and co-developing and sharing resources. On average, degree centralizations were higher than betweenness centralizations, meaning that while networks tended to have some agencies with substantially more connections than others, they were not dependent on a few agencies to bridge gaps to hold the networks together.

**Table 2.** Average degree^a^, degree centralization^b^, and betweenness centralization^c^ summarized over five service areas for five activity relationships.

	Average degree		Degree centralization		Betweenness centralization	
Activity	Mean	SD	Mean	SD	Mean	SD
Exchanging general information	11.7	2.3	0.454	0.069	0.132	0.064
Promoting ongoing services or programs	8.4	1.6	0.485	0.146	0.196	0.084
Annual/one-time events	6.6	2.3	0.418	0.093	0.230	0.116
Developing & sustaining ongoing services or programs	5.1	2.2	0.438	0.201	0.298	0.124
Developing & sharing resources	4.8	2.0	0.326	0.056	0.205	0.072

^a^Average number of connections for the agencies in the network.

^b^Extent to which the network has one or a few agencies with many connections.

^c^Extent to which the network has one or a few agencies that keep the network connected.

**Figure 1.**
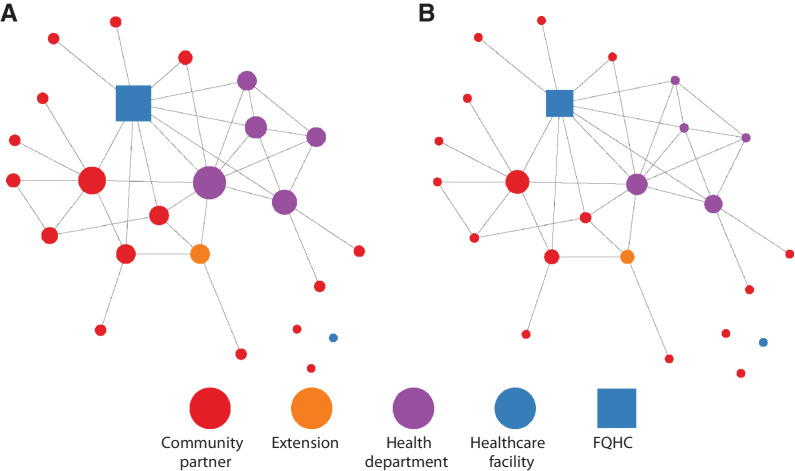
Area 3 sharing resources network. Agencies are sized by degree (**A**) and betweenness centrality (**B**).

Figure 1 shows one service area's network for sharing resources. Each node (circle or square) represents a different agency, with different colors representing the type of agency. The presence of a line (link) between two agencies indicates collaboration to develop and share resources. Figure 1 has two maps. In Panel A, the larger nodes indicate agencies with greater numbers of connections for sharing resources (degree). The larger nodes in Panel B highlight agencies that have a greater ability to serve as connectors to link agencies that are not directly connected to each other (betweenness centrality). In this example, the FQHC (square) served as a connector between several agencies that were not directly connected to each other, particularly the two community partners (red) that were only connected to the network through the FQHC. Three agencies were not connected, meaning they did not collaborate to develop and share resources with any other agencies. Maps with nodes sized by degree highlight agencies that were highly connected to other agencies. Maps with nodes sized by betweenness highlight agencies that can serve as connectors. The map also shows that for this service area, health departments (purple) were clustered together and developed/shared resources more with each other than with other kinds of agencies.

**Figure 2.**
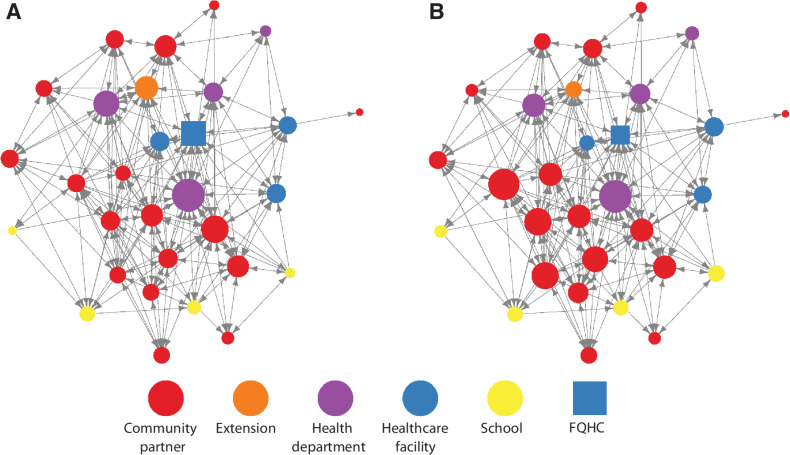
Area 1 referral network. Agencies are sized by in-degree (**A**) and out-degree (**B**).

Figure 2 shows a referral network from a different service area. The direction of the arrows represents where an agency received or sent referrals, and where a line has two arrows, it means the agencies both sent and received referrals to and from each other. Panel A sizes nodes by in-degree and highlights the agencies that received referrals from many other agencies. Panel B sizes nodes by out-degree and highlights agencies that sent referrals out to many other agencies.

### Uses of network findings

Rural agency staff who received the summary infographic and interactive Network Navigator with network figures for their area described multiple current, planned, and potential uses for the network information during navigator orientation sessions provided by the study team (Table 3). Agency staff (*n* = 14) described the usefulness of the network images and information for identifying gaps and planning which relationships to newly establish or enhance to strengthen their collaborative activities and cross-referrals. Staff also found the network information helpful to better understand the collaborative roles agencies had with each other. Several agencies have begun using network information to inform strategic planning, and had integrated network images and information in grant applications and reports to current funders to demonstrate collaboration strengths.

**Table 3.** Practitioner uses of network information.

Use	Audience	Description
Understand the network and its agencies	Agencies, network	To better understand:
		Roles the agencies have with each other
		Extent one's own agency is integrated in the network
		Extent of connectedness between agencies
		Whether perceptions of partnering match what agencies report
		The network to inform planning and intervention implementation
Show network strengths	Funders	Use in reports to funders
		Use in grant applications to:
		Show how well connected the agencies are
		Show resource needs so can get more resources in area
		Which partnerships are preexisting
Identify gaps	Agencies, network	Identify gaps in connections
		Identify gaps with specific partners
		Identify relationship building opportunities
		Show what to work on to improve partnering
Training	Staff, boards	Use in board trainings to show where can improve relationships
		Use as a training tool for new staff
Strengthen networks/improve collaborations	Existing partners	Forge greater relationships with existing partners
		Identify some partners need to engage with at a higher level
	New partners	Create new partnerships
		Learn where need growth to better align with mission
		Learn which activity types they can be more involved with
		Make strategic decisions about developing new connections
Improve referrals	Community Health Workers (CHWs), Patient Navigators	Identify which agencies community health workers (CHWs) to initiate or increase contact with
		Ensure each network agency is in CHW resource list
		Share network information with CHWs as a community resource
		Stay up to date on where to refer clients and for what
	Agencies	Ensure all needed memoranda of understanding are in place
		Support reimbursement for referrals, such as dietician referrals
Planning	Agencies, network	Use in agency's own strategic planning process
		Use in community assessments

## Discussion

Identifying informal multisector networks’ structures, strengths, and gaps through SNA can inform future informal or formal collaboration for cancer prevention and control (35, 46–49). Disseminating network findings via summary infographics and interactive platforms can enhance usefulness of SNA to practitioners in rural health and social service agencies. In their review of SNA in public health, Luke and Harris suggest such approaches should be utilized more frequently to communicate findings with public health agencies and communities (46). Public health practice increasingly recognizes the value of SNA, yet the use remains limited, especially in rural areas (34). While SNA is a common method to study formal coalitions and complex interventions in urban areas and report out to research audiences, it is less common to study rural networks, informal networks, or report how practitioners use network findings (50). In the present study of five rural service area informal networks in cancer prevention, rural agency staff found network images and statistics for collaborative activities helpful to demonstrate collaborative strengths in reports to funders and in grant applications, to identify gaps in connections, and plan ways to strengthen collaborations for health promotion and cancer prevention and control.

A network analysis of organizations in an urban community involved in an informal partnership for chronic disease prevention found a core of highly connected organizations, and a periphery of less connected organizations that had connections to core agencies but not to each other (35). The authors shared network graphics in a meeting with practitioners, noting one organization found it so useful they conducted a network analysis of a disease-specific collaboration with guidance from the researchers (35). In our study, the informal rural networks had a number of agencies with high ability to connect organizations not directly connected to each other. The rural networks did not rely on just a few agencies to bridge gaps. This is a strength, as when one agency is addressing a crisis, other agencies can keep the network well-connected to coimplement and promote ongoing cancer prevention and control efforts. While highly centralized networks that rely on a single hub agency may be more efficient (51), decentralized networks as found in the present study are less vulnerable to agency overwhelm (51).

The exchanging information relationship had a higher average degree than the more time- and resource-intensive activities. In an Australian city, researchers also found a high degree of information exchange and fewer connections for sharing resources and implementing joint programs (49). Held and colleagues (2021) found 48% of the organizations in an Australian urban informal network reported contributing resources to local chronic disease prevention efforts (35). An assessment of comprehensive cancer control programs in the United States found 58% reported coalition partners assisted with implementation of prevention interventions, with 62% reporting partners helped implement cancer screening (24). More study of rural networks and cross-sector referral networks is warranted, especially given the need to address social determinants of health so that cancer prevention and control efforts can be more effective (52, 53).

### Recommendations for practitioners

While there are no specific ideal values when comparing connectivity or centralization between networks, a network should be well-enough connected so that tasks are accomplished, but overly-saturated networks are a possible indication of redundant effort. While highly centralized networks are efficient, they are also vulnerable if the central agencies (or key individuals in central agencies) do not have the capacity to facilitate communication and collaboration between network partners. The more important issue is whether the appropriate agencies, in terms of expertise, mission, and capacity, are connected for the tasks at hand. This is precisely why knowledge of the network context from the practitioners within it is so important: those who are familiar with the network understand who should be connected. Practitioners and policy makers can use network maps in strategic planning, to mobilize communities to effectively implement interventions (48), and as an evaluation tool to assess whether an initiative successfully promoted and sustained increased collaboration (47).

Practitioners can use network information to demonstrate strengths, identify gaps, enhance existing collaborations, and build new relationships. We recommend organizations and networks reflect on their community health goals and priorities prior to engaging with network information, then review the network information to see if the partnerships needed to meet those goals are in place. Collaborators can ask for explanations of the images and network statistics, as well as access to hands-on training in how to use an interactive Network Navigator platform. We recommend that users start out with a high level view of the connections and then drill down into the nuances in order to build a rich understanding of how their organization interacts and connects with others and to identify areas that need improvement. Practitioners can determine their agency's connections, then look for connections not made and ask why.

Given limited resources in rural agencies (1), there is a need to understand assets and service capacity available within other organizations and leverage resources across networks to avoid depletion of any agency's capacity to provide services. Rural areas can seek outside assistance with social needs, such as transportation, housing, and disparities in food access, as there tend to be few resources within the area. For example, the only transportation resources in some rural service areas in the United States are small companies that can get Medicaid reimbursement or managed care companies that offer their own transportation, each of which have many stipulations and do not serve all the clients that need transportation support. In our interviews, stakeholders indicated transportation was a key barrier to cancer screening and treatment services among rural residents (36). Network visualizations and analyses can help communities identify resources to address social needs and disparities in modifiable cancer risk factors (53–55).

### Recommendations for researchers

We have several suggestions for researchers studying multisector collaboration in rural areas. It is useful to co-develop a network survey with agency staff, or at minimum, get agency staff input on a draft survey. Relationship dynamics exist inside the networks that are not evident to researchers from outside the area so it is useful to conduct initial sleuthing with local partners who can help identify agencies not on researchers’ initial lists, especially in rural areas without publicly available resource tracking systems. Due to the variety of resources and agencies, it is imperative to include all agencies with resources and maintain updated resource lists. Each rural area is unique; do not treat rural areas as if they are the same. Rural communities also vary in how they work together on health initiatives. It is useful to conduct pre–post network analyses to learn whether linkages are strengthened after a collaborative community health intervention. It is also helpful to compare how underresourced communities connect across organizations versus communities with more resources. One-time orientations to an interactive Network Navigator platform for users may be insufficient; instead, periodically offer one-on-one remote or in-person follow-up navigator use sessions after the initial orientation. To maximize usefulness of the network information, disseminate findings to participating agencies in a timely manner with minimal jargon and clear explanations so the information and included partners are current, accurate, and actionable.

### Limitations

Our study has some limitations. Some organizations were missed as network survey invitees, as a final list check was not feasible due to constraints health department staff faced during a global pandemic. Determining which agency staff were most familiar with the organization's collaborations was difficult, so the correct agency representatives may not have always been chosen to complete the survey. Regardless, this study is consistent in agency composition with a U.S. study with 162 public health networks where governmental and community-based organizations were predominantly in the health, education, and social service sectors (51). This study was cross-sectional, whereas there would be added value to conducting longitudinal network analyses (56). Recall bias is likely since we asked about collaboration in the previous calendar year because of agencies prioritizing responses to the COVID-19 pandemic in 2020 when data were collected. By the time we disseminated findings to participating agencies, new partners had been added in at least two service areas that were not included in the survey. Descriptions of the usefulness of the network application were limited to the context (partnerships and activities) for which they were designed. Despite these limitations, partners still found the network information valuable for reporting and planning purposes.

SNA is a useful tool for practitioners and researchers seeking to control cancer and other chronic conditions (35, 46–49, 57). Cross-sectional network analyses of multisector collaborations in health promotion/cancer prevention and control in rural areas can help partnering agencies identify network strengths and gaps, and point to ways to strengthen multisector collaboration. Disseminating network findings with rural health and social service agency staff through infographics and an interactive Network Navigator platform can enhance the usefulness of the information to practitioners. By identifying collaboration gaps, enhancing collaborative relationships, and planning collaboratively, underresourced rural areas can better leverage resources to coimplement evidence-based approaches to better address system-level risk factors (e.g., inadequate access to healthy foods), promote modifiable protective factors (e.g., physical activity), and increase access to early cancer detection (e.g., mammography screening; refs. 20–21, 23, 30, 32, 48, 58). To eliminate geographic disparities in modifiable cancer risk and protective factors, future study of the quality of information exchange and connection to external resources among complete informal and formal rural networks can inform ways to improve network effectiveness in risk factor modification.

## Supplementary Material

Supplementary Data
