# Supplementary material for: Mapping the Lay of the Land: Using Interactive Network Analytic Tools for Collaboration in Rural Cancer Prevention and Control
Source: Cancer Epidemiol Biomarkers Prev. 2022 Apr 20;31(6):1159–67. doi: 10.1158/1055-9965.EPI-21-1446 (PMC9167755; doi:10.1158/1055-9965.EPI-21-1446)
Supplement: Supplementary Data [file epi-21-1446_supplementary_methods_and_materials_suppsmm.docx]

Supplementary Methods and Materials: Social Network Survey

Q2 Instructions

The following questions will ask you about relationships and collaborations between ${e://Field/Organization} and other organizations. We ask that you think about these as they were during the *calendar year of 2019* (January 2019 – December 2019). We are asking about this time frame to get an idea of how things were for an easy-to-remember year-long time period before COVID-19.

Q3 Awareness/Contact

The following is a list of organizations participating in your area. (The survey is set up so that the name of your own organization will not appear for you.) On average, how often did ${e://Field/Organization} have direct *professional* contact (e.g., meetings, phone calls, emails, faxes, or letters) with each of the following organizations during *calendar year 2019?* (Do not count listservs or mass emails.) If you are comfortable answering for your agency as a whole, please do so. If not, you can answer for yourself/your work unit.

|  | Unaware of this organization (1) | Aware, but no contact (2) | Yearly (3) | Quarterly (4) | Monthly (5) | Weekly (6) |
| --- | --- | --- | --- | --- | --- | --- |
| Org1 (1) |  |  |  |  |  |  |
| Org2 (2) |  |  |  |  |  |  |
| Org3 (3) |  |  |  |  |  |  |
| Etc… |  |  |  |  |  |  |

Q4 Activities

With the organizations you indicated being in contact with, please indicate all of the activities ${e://Field/Organization} collaborated with them on during *calendar year 2019* [check all that apply]: We conceptualize "program" broadly. This could include ongoing clinical quality improvement, education programs, community policy, and other interventions.

|  | Annual/one-time events (e.g. planning, promotion, hosting, helping) (1) | Promoting ongoing services or programs (e.g. advertising, participant recruiting) (2) | Developing & sustaining ongoing services or programs (e.g. implementation, serve on each other's boards) (3) | Developing & sharing resources (e.g. grant writing, cost sharing, staff) (4) | Exchanging general information (e.g. updates, lessons learned, training materials) (5) | None of these (6) |
| --- | --- | --- | --- | --- | --- | --- |
| Org1 (1) |  |  |  |  |  |  |
| Org2 (2) |  |  |  |  |  |  |
| Org3 (3) |  |  |  |  |  |  |
| Etc… |  |  |  |  |  |  |

Q5 Referrals

Did ${e://Field/Organization} send or receive referrals with the following organizations during *calendar year 2019?* (Examples: screening services, physical activity or nutrition classes)

|  | We send referrals to them (1) | They send referrals to us (2) | Both send and receive (3) | Neither (4) |
| --- | --- | --- | --- | --- |
| Org 1 (1) |  |  |  |  |
| Org 2 (2) |  |  |  |  |
| Org 3 (3) |  |  |  |  |
| Etc… |  |  |  |  |

Q6 Facilitators

Please indicate your level of agreement that ${e://Field/Organization} experienced each of the following *facilitators* to collaboration with other organizations during *calendar year 2019:*

|  | Strongly Disagree (1) | Somewhat Disagree (2) | Neither agree nor disagree (3) | Somewhat Agree (4) | Strongly Agree (5) |
| --- | --- | --- | --- | --- | --- |
| Important issues to work together on (1) |  |  |  |  |  |
| Mutual willingness to help (2) |  |  |  |  |  |
| Previously-existing relationships (3) |  |  |  |  |  |
| Small community structure, sense of community cohesion (4) |  |  |  |  |  |
| Funding/resource sharing (5) |  |  |  |  |  |

Q7 Barriers

Please indicate your level of agreement that ${e://Field/Organization} experienced each of the following *barriers or challenges* to collaboration with other organizations during *calendar year 2019:*

|  | Strongly Disagree (1) | Somewhat Disagree (2) | Neither agree nor disagree (3) | Somewhat Agree (4) | Strongly Agree (5) |
| --- | --- | --- | --- | --- | --- |
| Competition for funding (prevention programming, outreach, or other activities) (1) |  |  |  |  |  |
| Competition for clients or unwillingness of others to collaborate (2) |  |  |  |  |  |
| Travel and transportation (distance, time, cost) (3) |  |  |  |  |  |
| Staff turnover and difficulty hiring and training staff (4) |  |  |  |  |  |
| Lack of time to attend meetings & events or offer services collaboratively (5) |  |  |  |  |  |
| Competing priorities (6) |  |  |  |  |  |
| Organization scope restrictions or perceived typical activities (7) |  |  |  |  |  |
| Leadership does not prioritize collaboration (8) |  |  |  |  |  |
| Unsure of who to contact (9) |  |  |  |  |  |

Q8 Desired Collaborations

Please indicate the level of interest ${e://Field/Organization} would have in working with other agencies on...

|  | Not at all Interested (1) | Somewhat Interested (2) | Very Interested (3) |
| --- | --- | --- | --- |
| Funding and/or providing low-cost preventive services for those with financial strain (e.g. uninsured, low income) (1) |  |  |  |
| Increasing local/nearby cancer screening or other preventive service availability (2) |  |  |  |
| Improving referral systems (3) |  |  |  |
| Collaborative grant writing (4) |  |  |  |
| Ongoing health promotion programs (5) |  |  |  |
| Community outreach, given no single organization in our area has enough staff for all the desired outreach (6) |  |  |  |

Q9 Given the topics discussed above, are there additional thoughts or comments you would like to add (e.g. about barriers, facilitators, desired collaborations, changes due to COVID)?

________________________________________________________________

________________________________________________________________

________________________________________________________________

________________________________________________________________

________________________________________________________________
